# Association between mental health conditions and rehospitalization, mortality, and functional outcomes in patients with stroke following inpatient rehabilitation

**DOI:** 10.1186/1472-6963-11-311

**Published:** 2011-11-15

**Authors:** Almas Dossa, Mark E Glickman, Dan Berlowitz

**Affiliations:** 1 Center for Health Quality, Outcomes, and Economic Research, ENRM VA Hospital, Bedford, MA, USA; 2 Boston University School of Public Health, Boston, MA, USA

## Abstract

**Background:**

Limited evidence exists regarding the association of pre-existing mental health conditions in patients with stroke and stroke outcomes such as rehospitalization, mortality, and function. We examined the association between mental health conditions and rehospitalization, mortality, and functional outcomes in patients with stroke following inpatient rehabilitation.

**Methods:**

Our observational study used the 2001 VA Integrated Stroke Outcomes database of 2162 patients with stroke who underwent rehabilitation at a Veterans Affairs Medical Center.

Separate models were fit to our outcome measures that included 6-month rehospitalization or death, 6-month mortality post-discharge, and functional outcomes post inpatient rehabilitation as a function of number and type of mental health conditions. The models controlled for patient socio-demographics, length of stay, functional status, and rehabilitation setting.

**Results:**

Patients had an average age of 68 years. Patients with stroke and two or more mental health conditions were more likely to be readmitted or die compared to patients with no conditions (OR: 1.44, p = 0.04). Depression and anxiety were associated with a greater likelihood of rehospitalization or death (OR: 1.33, p = 0.04; OR:1.47, p = 0.03). Patients with anxiety were more likely to die at six months (OR: 2.49, p = 0.001).

**Conclusions:**

Patients with stroke with pre-existing mental health conditions may need additional psychotherapy interventions, which may potentially improve stroke outcomes post-hospitalization.

## Background

Stroke is the third leading cause of death and a leading cause of adult disability [1]. Compared to other medical diagnoses, stroke has a higher mortality rate, more readmissions, and higher costs of care [2]. Patients without stroke, but having mental health disorders are also more likely to be re-hospitalized, have higher mortality rates, and have lower functional outcomes compared to patients without these disorders [3-9]. Moreover, when mental health disorders co-occur with other medical conditions, this co-occurrence tends to reduce quality of life, mortality, and adherence to interventions [10-14]. Although studies exist on patients with post-stroke depression and its association with readmissions, mortality, and functional outcomes [15-19], few studies have examined these outcomes in patients with stroke and pre-existing mental health disorders. Additionally, outcomes for patients with stroke and pre-existing mental health disorders may differ from outcomes for patients with post-stroke depression.

While limited evidence exists regarding pre-existing mental health disorders in patients with stroke and stroke outcomes, there is significant research showing that mental health conditions play an important role in outcomes such as readmissions, mortality, and functional outcomes. Research on elderly medical patients with mental health disorders showed that those patients with more than one psychiatric diagnosis had greater risk of rehospitalization [20]. Additionally, all forms of mental health disorders in medical patients including depression, substance abuse, psychosis, depression, bipolar disorder, anxiety disorder, and other mental health disorders were associated with the risk of readmissions at six months and one year [6,20]. Other studies showed increased mortality and rehospitalization for medical patients with major depression and for patients with post-stroke depression [5,21,19]. The relationship between depression and disability has also been well established [22,10,11]. Depression was significantly associated with decreased function from admission to discharge in a sample of older adults in sub-acute care, for patients with post-stroke depression at stroke onset and after six months, and for patients with stroke undergoing out-patient rehabilitation [15,18].

Patients with mental health disorders and medical illness may have poorer treatment adherence, are less motivated to seek care for their medical illness, have less access to health care, and may be more neglectful of their self-care management and health care needs [10,23,24]. They may also be less optimistic and enthusiastic about their rehabilitation regimen. Thus, patients with stroke and with the added burden of pre-existing mental health disorders may have worse outcomes such as increase in likelihood of mortality, hospital readmission, and worse functional outcomes than those without mental health disorders. Additionally, they may also have greater service needs. Examining the association between pre-existing mental health disorders and stroke outcomes such as readmissions, mortality, and functional outcomes in patients with stroke may have important implications for patient care. Additionally, knowing which specific mental health disorder is associated with these stroke outcomes may assist mental health clinicians to treat the particular condition proactively. More research is needed in this area in order to address the challenge of treating these complex patients.

To increase our understanding of the association between presence of any mental health condition, number of mental health conditions, and types of conditions and stroke outcomes of rehospitalization, mortality, and functional outcomes among patients with stroke, we addressed the following questions:

1. Is presence of any mental health condition compared to no condition prior to stroke associated with greater likelihood of post-discharge six-month rehospitalization and six-month mortality, and worse discharge functional outcomes in patients with stroke?

2. Is presence of one mental health condition compared to no condition, and more than one condition compared to no condition prior to stroke associated with greater likelihood of post-discharge six-month rehospitalization and six-month mortality, and worse discharge functional outcomes in patients with stroke?

3. Is the type of mental health condition associated with greater likelihood of post-discharge six-month rehospitalization and six-month mortality, and worse discharge functional outcomes in patients with stroke?

## Methods

### Sample and Database

Our study sample consisted of a national cohort of 2162 patients with stroke admitted between October 1, 2000 to September 30, 2001, who underwent inpatient rehabilitation at a Department of Veterans Affairs (VA) medical center. Patients could receive rehabilitation services at an acute care hospital, a sub-acute unit, or a long-term care unit. Time of onset of stroke to the rehabilitation admit date was no more than 30 days. We identified patients through their presence in the 2001 VA Integrated Stroke Outcomes Database (ISOD) from data that was used in a prior study [25]. This study was approved by the Bedford VA Institutional Review Board. The ISOD contains clinical and administrative information on veteran patients identified by a clinician as having a stroke. Included in the ISOD data base are the following: a) inpatient and outpatient diagnostic data from the National Patient Care Database, which provides data on demographics, diagnoses, procedures, and utilization from each Veterans' Affairs Medical Center, and contains information on all VA inpatient and outpatient episodes of care by fiscal year and location of care, b) mortality data from the Beneficiary Identification and Records Locator Subsystem, an administrative database that contains information on dates of death of all VA beneficiaries, and c) information about the rehabilitation stay from the Functional Status Outcomes Database (FSOD) such as patient demographics, diagnoses, discharge setting, length of stay, and admission and discharge functional outcome information.

The FSOD contains the VA portion of the Uniform Data System for Medical Rehabilitation Database, which is the most widely used data for assessing rehabilitation outcomes [26,27], and is collected by rehabilitation providers at patient admission and discharge. The VA inpatient rehabilitation program offers a team approach to the care of Veterans with physiatry, physical therapy, occupational therapy, and speech therapy services in order to achieve an optimal level of function and independence. The FSOD tracks information for all VA inpatient rehabilitation patients and is used to monitor the quality of rehabilitation care delivered to Veterans. Studies have reported the reliability and validity of the component data bases comprising the ISOD [28,29].

**Figure 1:**
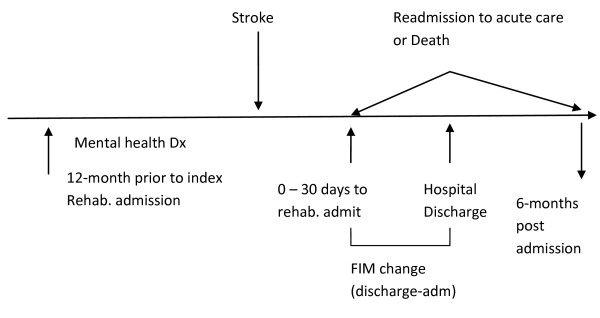
**Time order of events before and after stroke admission**.

Figure 1 is a graphic representation that shows the time order of events for the cohort including the relationship of the incident stroke to the retrospective period, to the mental health diagnoses, and to the outcome variables.

### Outcome Measures

Our three outcome measures included rehospitalization/death, mortality, and change in functional outcome. We defined our first outcome measure as a binary indicator of six-month rehospitalization or death (henceforth "rehospitalization/death"), versus alive and not rehospitalized within six-months of the inpatient rehabilitation admission. The rationale for this measure is that by using rehospitalization alone, death would be a censoring event if it were to occur within six months after rehabilitation discharge. We specifically considered rehospitalization to have occurred when a patient was readmitted to an acute medical-surgical unit within a six-month period following the admission to the hospital for stroke. The patient could be re-admitted either from home or from a rehabilitation or long-term care unit. Readmissions included all cause readmissions. For six-month mortality, we used a binary indicator. We considered six-month mortality as mortality within a six-month period following the admission to the hospital for stroke. For our third outcome of interest, functional outcome, we used change in functional independence measure (FIM) score from admission to discharge during the hospital stay. Physical and occupational therapists measure the FIM score at initiation of rehabilitation and at discharge, which includes scores on a standardized measure of basic daily living skills [30]. The FIM is an 18-item ordinal scale with 13 motor items and five cognitive items used with the rehabilitation population and is a useful assessment of the patient's progress during inpatient rehabilitation. The items evaluate the patient's ability in self-care such as eating, grooming, and bathing, mobility such as transfer skills, locomotion skills, sphincter control, and skills such as social interaction, problem solving, and memory. For each item, the seven point Likert scale ranges between being totally dependent to independent. The total score can range from 18 to126, with higher scores indicating better functioning. We computed this change score as the difference between discharge FIM and admission FIM.

### Independent Measures

#### Mental health Conditions

We followed the same framework used by Frayne and colleagues who developed a valid system of identifying mental illness from patient administrative records [31]. Frayne drew from the conceptual framework developed by a panel of experts for the American Psychiatric Association's Diagnostic and Statistical Manual of Mental Disorders, Primary Care, (DSM - IV-PC) fourth edition, which identified broad clusters of mental health conditions seen in primary care [32]. The DSM-IV-PC uses a descriptive approach, i.e. identification of symptoms and development of diagnostic algorithms that are organized by symptoms, and emphasizes only those conditions regularly present in primary care [33]. For example, the condition "depressed mood" includes a range of psychiatric conditions such as major depressive disorder, bipolar I disorder currently depressed, adjustment disorder currently depressed, adjustment disorder with depressed mood, and depressive disorder not otherwise specified [31]. For a primary care provider, patients in this cluster would present with a somewhat similar clinical appearance. Thus, although the DSM -IV -PC explicitly maps to ICD-9 codes, it has a clinical focus in order to allow primary care providers to recognize classes of psychiatric conditions. In order to apply this framework for their needs, Frayne and colleagues had an expert panel of practicing internists review the full list of the DSM-IV-PC conditions and modified this list to end up with a set of ten primary mental health conditions, which they called: depressive disorder, anxiety, psychotic symptoms, manic symptoms, problematic substance abuse, dysfunctional personality traits, dissociative symptoms, somatoform symptoms, impulse control disorders, and eating disorders.

Therefore, the mental health conditions in our study included the same conditions. Our main categories included depression, anxiety, psychotic conditions, and substance abuse disorder. However, we also included another category "Other mental health conditions", since the other disorders (manic symptoms, dysfunctional personality traits, dissociative symptoms, somatoform symptoms, impulse control disorders, and eating disorders) equaled a total of 2.82%, and each condition represented less than 1% of the sample. For our study, no overlapping ICD-9 codes existed among the 10 mental health conditions. For our mental health conditions, we used inpatient and outpatient diagnoses and both primary and secondary diagnoses from the National Patient Care Database for the year before admission. The most widely used typology for classifying mental health conditions by VA practitioners is the DSM-IV-PC, which links explicitly to ICD-9 codes. Diagnoses are recorded at every visit.

#### Control variables

Variables potentially affecting the outcomes included age, gender, race/ethnicity, marital status, functional status, marital status, length of stay, and co-morbidities [16,19,34-46]. As noted in other research [35,40,47], another potential predictor of short-term mortality and rehospitalization was discharge functional status. We used discharge FIM for this control variable. For the FIM change score outcome, the admission FIM score was used as a control variable [44]. Other independent variables incorporated into the FIM change model included admission care setting [38,41], which included mutually exclusive categories of acute rehabilitation setting versus other settings such as sub-acute setting, and continuum of care setting (when patients transition across acute care, sub-acute and long-term care). Additionally, we included admission rehabilitation class, which included mutually exclusive categories of initial rehabilitation versus other (continuing rehabilitation, readmission, short stay evaluation, and unplanned discharge). Race/ethnicity was a binary variable, which only included White (Caucasian) and Non-White categories. Age was modeled as a linear effect. We used the Charlson index to measure co-morbidities. This index scores each condition by weighting them on the basis of their association with one-year mortality [48], and was developed and validated originally for a cohort of breast cancer patients. Although a variety of co-morbidity measures exist, we selected the Charlson index as it is widely understood and most commonly used. It has been used subsequently in stroke outcome and functional outcome rehabilitation studies [17,49,50]. For our study, we excluded cerebrovascular disease from the Charlson index [49].

### Analyses

We computed descriptive statistics for the clinical, socio-demographic, and utilization variables. We calculated mean values or percentages for variables for patients with and without mental health diagnoses along with bivariate analyses to measure group differences for sociodemographic and clinical variables. We also analyzed our independent variables for multicollinearity by computing variance inflation factors for each predictor variable, coding each k-level categorical variable as a set of k-1 binary indicators. We conducted bivariate analyses between our outcome variables and mental health conditions.

Our multivariate analyses included logistic regression models for the outcomes 6-month rehospitalization/death and 6-month mortality, and linear regression models for the FIM change score. Our first model examined the association between any mental health condition and stroke outcomes. To study the effects of the number of mental health conditions on stroke outcomes, our second model included mental health conditions as a three-level categorical variable (coded as two binary indicators) with levels for no condition, one condition, and more than one condition. The decision to categorize our mental health conditions in this manner was based in part on the low numbers of patients with more than one MH condition. To examine the effect of different mental health conditions, we conducted two types of analyses for the third model: a) Fit models that included all of the mental health conditions in order to assess the significance of each condition beyond the effect of the other conditions, and b) fit separate regressions where each mental health condition was included without the others in the models in order to assess the individual effect of each condition.

To address the modest amount of missing data, we used multiple imputation on the set of independent variables. Five sets of imputations were generated using Monte Carlo Markov chain simulation from an approximating multivariate normal distribution of the predictors. Coefficient estimates and standard errors were constructed using usual multiple imputation combination rules [51], and compared the fit of the models constructed using the imputed data sets with the models fit on the complete-case data. Our final models presented below rely on cases with the imputed data on all predictor variables. We used SAS version 9.1 to perform the analyses.

## Results

### Sample characteristics

Our data set included 2162 patients with stroke receiving inpatient rehabilitation at a VA facility from October 1, 2000 to September 30, 2001. The stroke onset to admit rehabilitation date varied from 0 to 30 days with a mean of 6 days. Patients had an average age of about 68 years, were predominantly male, approximately two-thirds were white, and about half were married. They showed a moderate degree of baseline functional impairment. An analysis of variance inflation factors revealed that none of the independent variables showed multicollinearity. Our highest variation inflation factor was 3.49, which is less than 10, the value often considered the threshold over which collinearity is considered a concern.

Table 1 shows descriptive data on socio-demographic, clinical variables, and outcome variables, and differences in independent variables for patients with and without mental health conditions. Our bivariate analyses showed that patients with mental health conditions were more likely to be younger (p < 0.0001), unmarried (p = 0.007), have a longer length of stay (p = 0.006), and have lower admission FIM scores (p = 0.03). Ninety three percent of patients underwent an initial rehabilitation stay. Fifteen percent of the patients were admitted to acute rehabilitation settings, 5% of patients were admitted to sub-acute rehabilitation settings, and 80% of the patients were admitted into a continuum of care setting. Eighty three percent of the patients had ischemic strokes and 6% had hemorrhagic strokes, the rest had unspecific cerebrovascular disease. The rehabilitation length of stay was about 22 days.

**Table 1:** Socio-demographic and clinical characteristics

Characteristics	n (%) or Mean ± SD	Mental Health	No Mental
(Total N)		Conditions	Health
		n (%) or ± SD	Conditions
Age (2162)	68.21 ± 11.06	65.65 ± 11.62	69.51 ± 10.54
Length of stay (2162)	22.29 ± 22.29	24.17 ± 24.67	21.02 ± 20.51
Admission FIM score (2162)	68.38 ± 29.95	66.16 ± 29.30	69.38 ± 30.28
Discharge FIM score (2094)	87.80 ± 32.18	86.39 ± 31.48	88.14 ± 32.54
Change FIM score (2089)	18.93 ± 19.16	19.53 ± 19.76	18.38 ± 18.86
Charlson Index (2077)	1.79 ± 1.99	1.91 ± 2.09	1.75 ± 1.95
**Race/Ethnicity(2133)**			
White	1412 (66.20)	393 (19.18)	961 (46.90)
Non-White	721 (33.80)	177 (8.64)	518 (25.28)
**Marital Status (2089)**			
Married	1011(48.40)	245(12.21)	734(36.59)
Unmarried	1078(51.60)	313(15.60)	714(35.50)
**Gender (2112)**			
Male	2075(98.25)	552(27.13)	1446(71.06)
Female	37 (1.75)	12 (0.59)	25 (1.23)
**Outcomes**			
Rehospitalization (2117)	574 (27.11)		
Rehospitalization/death (2117)	726 (33.58)		
Mortality (2117)	256 (12.09)		
Change FIM score (2089)	18.93 ± 19.16		

Table 2 shows the distribution of mental health conditions and frequency of number of mental health conditions. Twenty eight percent of all patients were diagnosed with mental health conditions. Out of these patients, 15.55% had a mental health condition of depression, 8.67% had an anxiety condition, 5.73% had a psychotic condition, 7.41% had a substance abuse condition, and 2.82% had other mental health conditions (unexplained physical symptoms, impulse control disorders, manic disorders, and eating disorders). About 4% of patients had both depression and anxiety.

**Table 2:** Estimates of pre-stroke mental health conditions

Mental Health Conditions	N (%)
**Any mental health condition**	578 (27.83)
One mental health condition	389 (18.73)
Two mental health conditions	138 (6.64)
More than two mental health conditions	51 (2.46)
Types of mental health conditions *	
Depression	323 (15.55)
Anxiety	180 (8.67)
Psychotic symptoms	119 (5.73)
Substance abuse disorder	154 (7.41)
Other mental health conditions	61 (2.82)
Depression and anxiety	88 (4.24)

*Raw numbers sum up to more than 578 because some veterans had more than one mental health disorder

### Six-month rehospitalization/death

Bivariate analyses showed that presence of more than one mental health condition was significantly associated with six-month rehospitalization/death (OR: 1.34, p = 0.04). Our logistic regression model did not find a significant association between any mental health condition and rehospitalization/death. In examining the association of number of mental health conditions, and after adjusting for control variables, our logistic regression model (Table 3), showed that the presence of one mental health condition was not significant (no mental health condition as reference), but the presence of more than one mental health condition was significantly associated with six-month rehospitalization/death (OR: 1.44, p = 0.04).

**Table 3:** Logistic regression for mental health conditions and six-month rehospitalization/death

Variables	OR (CI)	p value
One mental health condition	1.13 (0.88, 1.47)	0.34
> 1 mental health condition	1.44 (1.02, 2.04)	0.04
(ref: no mental health condition)		
Discharge FIM score	0.99 (0.98, 0.99)	< 0.0001
Charlson Index	1.21 (1.15, 1.27)	< 0.0001
Race/Ethnicity (White)	0.99 (0.81, 1.22)	0.95
Married	.99 (0.81, 1.20)	0.90
Length of stay	1.00 (0.99, 1.00)	0.07
Age	1.01 (1.00, 1.02)	0.01
Male gender	1.005(0.49, 2.23)	0.91

N = 2,049

Bivariate analyses between type of mental health condition and rehospitalization/death showed that depression was significantly associated with rehospitalization/death (OR: 1.40, p < 0.008). Our logistic regression models (Table 4) show the association between types of mental health conditions and six-month rehospitalization/death. Both depression (Model I) and anxiety (Model II) were significantly associated with six-month rehospitalization/death only in the models that included depression and anxiety without controlling for the other mental health conditions (OR: 1.33, p = 0.04; OR: 1.47, p = 0.03, respectively).

**Table 4:** Logistic regression models for depression and anxiety and six-month rehospitalization/death

	Model I Depression		Model II Anxiety	
Variables	OR (CI)	p value	OR (CI)	p value
Depression	1.33 (1.02, 1.75)	0.04		
Anxiety			1.47 (1.04,2.07)	0.03
Discharge FIM score	0.99 (0.98, 0.99)	< 0.0001	0.99 (0.98, 0.99)	< 0.0001
Charlson Index	1.20 (1.15, 1.23)	< 0.0001	1.22 (1.16, 1.28)	< 0.0001
Race/Ethnicity (White)	0.99 (0.80, 1.22	0.90	0.99 (0.81, 1.23)	0.98
Married	0.98 (0.80, 1.20)	0.85	0.99 (0.81, 1.21)	0.91
Length of stay	1.00 (0.99, 1.00)	0.09	1.00 (.99, 1.00)	0.08
Age	1.01 (1.00, 1.02)	0.02	1.01 (1.00, 1.02)	0.02
Male gender	1.06 (0.49, 2.29)	0.98	1.05 (0.49, 2.27)	0.90

N = 2,049

### Six-month Mortality

We did not find significant effects for any mental health condition and the number of mental health conditions on mortality. Our bivariate analyses between type of mental health condition and six-month mortality showed that anxiety was significantly associated with six-month mortality (OR: 1.72, p = 0.02). In examining the association between types of mental health conditions and mortality, our regression model showed that the presence of an anxiety condition was significantly associated with patients dying in the six-month period when controlling for the other mental health conditions (Table 5, OR: 2.49, p = 0.001). In an additional analysis, in which each mental health condition was included without the others, the results were similar, i.e. anxiety was significant (OR: 2.39, p = 0.001, table not shown).

**Table 5:** Logistic regression for mental health conditions and six-month mortality

Variables	OR (CI)	p value
Depression	0.98 (0.61, 1.58)	0.94
Anxiety	2.49 (1.42, 4.34)	0.001
Psychosis	1.13 (0.58, 2.19)	0.72
Substance Abuse	0.56 (0.24, 1.32)	0.19
Other Mental Health Conditions	0.86 (0.28, 2.63)	0.79
Discharge FIM score	0.97 (0.96, 0.97)	< 0.0001
Charlson Index	1.24 (1.15, 1.33)	< 0.0001
Race/Ethnicity (White)	1.28 (0.88, 1.89)	0.20
Married	1.04 (0.74, 1.47)	0.81
Length of stay	1.00 (0.99, 1.01)	0.23
Age	1.02 (1.00, 1.03)	0.07
Male gender	2.73 (0.34, 21.93)	0.34

N = 2,049

### FIM Change Score

We did not find significant associations between any mental health condition and number of mental health conditions and FIM change score. Our bivariate analysis showed that anxiety was significantly associated with FIM change score (estimate: 3.31, p = 0.03). Although not significant at a p level of 0.05 level, our model (Table 6) showed that patients specifically with anxiety and patients in the category of "other mental health conditions" showed functional outcome changes at discharge when controlling for the other variables at a 0.1 significance level. Table 6 shows that for patients with anxiety the FIM change estimate increased by 2.63 points compared to patients without anxiety disorder (p = 0.07). However, for patients in the category of "other mental health disorders", the FIM change estimate decreased by 4.27 points compared to patients without these disorders (p = 0.08). In our additional analysis, in which each mental health condition was included without the other mental health condition, anxiety was not significant, and other mental health disorders was significant at a 0.1 level (FIM change estimate: -4.20, p = 0.07).

**Table 6:** Linear regression for different mental health conditions and FIM change score

Variables	Coeff (SE)	p value
Intercept	35.88 (4.42)	<.0001
Depression	-0.16 (1.13)	0.89
Anxiety	2.63 (1.46)	0.07
Psychosis	-1.55 (1.73)	0.50
Substance Abuse	- 1.07 (1.52)	0.48
Other Mental Health	-4.27 (2.40)	0.08
Conditions		
Charlson Index	-0.73 (0.20)	0.0002
Admission FIM score	-0.09 (0.01)	< 0.0001
Race/Ethnicity (White)	1.77 (0.81)	0.03
Length of stay	0.27 (0.02)	< 0.0001
Initial Rehabilitation stay	3.13 (1.52)	0.04
(ref: Other)		
Acute rehabilitation setting	5.40 (1.04)	< 0.0001
(ref: Other.)		
Age	-0.22 (0.04)	< 0.0001
Male gender	-4.19 (2.92)	0.15

N = 2,049

### Sensitivity to Missing Data

The results of the multiple imputation analyses were similar to complete-case analyses in which observations were removed if any of the independent variables had missing data; the coefficient estimates were only slightly different, and the significance of the predictors at the 0.05 level were the same in each instance as in the multiple imputed analyses.

## Discussion

This is the first study examining the association of a broad range of pre-existing mental health conditions and rehospitalizations, mortality, and functional outcomes for patients with stroke undergoing inpatient rehabilitation. Our findings showed that the presence of two or more mental health conditions in patients with stroke was significantly associated with the rehospitalization/death outcome variable at six-months compared to patients with no mental health conditions. Depression and anxiety were significant for the rehospitalization/death outcome at six-months. Additionally, anxiety was significantly associated with the mortality outcome at six-months. However, patients with an anxiety disorder showed a trend towards short-term increased functional outcome improvement at discharge compared to patients without anxiety. Patients with "other mental health conditions" showed a trend towards decreased functional outcome improvement at discharge compared to patients without "other mental health conditions".

Consistent with other studies that have shown that mental health conditions can increase hospitalizations in patients with stroke and in patients with other medical diagnoses [5,7,8,17], we found that rehospitalization/death was significantly associated with having two or more mental health conditions. In our study of patients with stroke, depression was associated with rehospitalization, which is supported by the study on patients with post-stroke depression [21]. Although we did not find other studies on post-stroke anxiety and rehospitalization, other studies on older medical patients have shown that depression and anxiety disorder are both associated with rehospitalization [5,20,21]. One possible explanation for increased rehospitalization in our study is that it may be more difficult to stabilize patients with mental health conditions. They may not adhere to medication and self-management regimens [23]. Health professionals need to be alert to the risks of readmissions for patients with stroke and co-occurring mental health conditions, and need to investigate types of outpatient services that would prevent readmissions in this population. Further research on medications and other types of services is needed for patients with stroke with mental health disorders. Reducing avoidable hospital readmissions can help to reduce health care costs and improve quality of care. Often, the focus is on home or outpatient rehabilitative care for mobility disorders, but a focus on outpatient mental health follow-up may benefit these patients and prevent readmissions.

Our findings on increased mortality are supported by a study that showed that elderly patients with mental health disorders had higher rates of mortality when compared to those without mental health disorders [52]. Although other studies have reported on patients with post-stroke mental health disorders and association with increased mortality compared to patients without mental health disorders [16], our study reported specifically on the association of increased mortality and pre-existing anxiety disorder. Our study also showed that despite the fact that patients with mental health conditions were younger, their mortality risk and readmission rate was still higher than those patients without mental health conditions. This finding is consistent with findings from another study in veterans with post-stroke depression and other mental health diagnoses such as schizophrenia, anxiety disorders, personality disorders, and substance abuse who were more likely to die despite the fact that they were younger [19].

Our finding that patients with anxiety tended to show increased short-term functional outcome improvement at discharge was surprising, and we were unable to find other studies that support this finding. Possibly, patients with anxiety tend to be more concerned about their functional issues and subsequently participate more actively in inpatient rehabilitation. We did not find any associations between depression and functional outcomes. Our findings differ from other studies that show a significant association between post-stroke depression and functional outcome limitation and between depression and functional outcome in patients with other medical problems [9,15,53]. In another study on patients with stroke receiving sub-acute rehabilitation, minor depressive symptoms diagnosed within five days of admission were significantly associated with a decreased FIM score change from admission to discharge, but not from discharge to 90-day follow-up [15]. Clearly, the association of depression and functional outcomes is an important area needing further study. A possible reason for the lack of association between functional outcomes and patients with pre-existing depression could be because patients with stroke accepted into inpatient rehabilitation may be less likely to have severe mental health disorders. We did not have information on the severity of mental health conditions and it is possible that the more severe disorders are associated with decreased functional outcomes. However, it is possible that having more than one mental health condition could be a surrogate for severity. The patients in our study may also be better controlled with medications, since they had pre-existing mental health conditions compared to the patients with post-stroke depression in other studies. Future studies could examine the association between severity of disorder and functional outcomes. Further, our study only examined the association between mental health conditions and short-term inpatient functional outcomes at discharge, and future studies need to examine the association between long-term functional outcomes and mental health conditions in patients with mobility impairments.

This study has limitations. First, we did not have information on patients who developed mental health conditions post-stroke, and it is possible that within this six-month period other patients did develop post-stroke mental health disorders, which could potentially affect our results. Future studies could examine the effect of pre-existing mental health diagnoses compared to post-stroke mental health diagnoses and its association with stroke outcomes. Secondly, we used administrative data and were therefore limited in our choice of control variables. For example, we did not have information on socio-economic issues or presence of an informal caregiver, which may be associated with hospital admissions. Additionally, our dataset did not have detailed racial-ethnic information. Other limitations in using administrative data include the fact that mood disorders and other mental health problems are frequently under-diagnosed and undertreated in older adults [54]. They may also be transitory in nature. Additionally, diagnostic information comes from billing codes used in administrative data, which likely are less reliable than information from structured clinical interviews or rating scales. Thirdly, we did not have information on the severity of the stroke diagnoses, which could potentially impact the stroke outcomes we measured. Fourthly, although we studied the association of the number of mental health conditions with stroke outcomes, one cannot assume that they had an additive affect on the outcome variables. We were unable to study comorbidity patterns (such as anxiety and depression) due to lack of sufficient power. Fifth, we use the Frayne classification to develop our mental health conditions, using the DSM-IV-PC categories, which differ from mental health diagnostic categories used in other studies. Therefore, our results need to be interpreted with caution. Lastly, we can only generalize this study to older patients with stroke who received inpatient rehabilitation, and to males since our study included a predominantly male population. Our findings need to be confirmed by other studies, and future research should target patients with different medical co-morbidities and mental health disorders in acute care rehabilitation as well as other settings such as sub-acute care and nursing homes. Finally, our data was observational and we are unable to draw causal conclusions from the findings of this study.

## Conclusions

A high percentage of patients with stroke have mental health conditions, and our findings suggest that the presence of mental health conditions are significantly associated with important stroke outcomes for patients who have received inpatient stroke rehabilitation. These findings have important implications for clinicians and researchers for improving care of patients with stroke. Researchers examining readmissions, mortality, and functional outcomes for patients with stroke need to consider their pre-existing mental health conditions. Clinicians treating patients with stroke need to prospectively identify specific patients with stroke with pre-existing mental health conditions for whom additional psychotherapy interventions may result in improved stroke outcomes. Further study may confirm that both behavioral as well as rehabilitation interventions and modifications specifically geared towards these disorders may help to prevent readmissions, deaths, and increase functional outcomes.

## List of Abbreviations

DSM-IV-PC: Diagnostic and Statistical Manual of Mental Disorders, Primary Care; FIM: Functional Independence Measure; FSOD: Functional Status Outcome Database; ISOD: Integrated Stroke Outcome Database; VA: Veterans Affairs; Note: ICD-9 was not spelled out as it is a common abbreviation

## Competing interests

The authors declare that they have no competing interests.

## Authors' contributions

AD contributed towards the research idea, design of study, analysis, and manuscript writing. MG contributed towards guiding the statistical analyses and overall manuscript writing, specifically the methodology and results sections. DB assisted with the design of the study, database information and set-up, analysis, and overall manuscript writing. All authors read and approved the final manuscript.

## Authors' information

AD has worked as a physical therapist in a variety of settings including acute care, rehabilitation, and home care. Her research interests include improving health care delivery and outcomes for patients with stroke.

## Pre-publication history

The pre-publication history for this paper can be accessed here:

http://www.biomedcentral.com/1472-6963/11/311/prepub
